# Cost of investigations during the acute hospital stay following total hip or knee arthroplasty, by complication status

**DOI:** 10.1186/s12913-020-05892-1

**Published:** 2020-11-12

**Authors:** Emma Cheng, Adriane Lewin, Tim Churches, Ian A Harris, Justine Naylor

**Affiliations:** 1 South Western Sydney Clinical School, South West Sydney Clinical School UNSW, Liverpool Hospital, Locked Bag 7103, Liverpool BC, NSW 1871 Australia; 2 Ingham Institute for Applied Medical Research, 1 Campbell St, Liverpool, NSW 2170 Australia; 3 Whitlam Orthopaedic Research Centre, Level 2, 1 Campbell St, Liverpool, NSW 2170 Australia

**Keywords:** Total hip arthroplasty, Total knee arthroplasty, Cost, Investigations, Imaging, Pathology, Complications, Post-operative complications

## Abstract

**Background:**

Total hip and total knee arthroplasties are among the most common types of surgery performed in Australia today and are effective treatments for severe osteoarthritis. However, the increasing financial burden on the health system owing to the increasing rates of surgery has led to a growing interest in improving the cost-effectiveness and safety of arthroplasty care. This study was designed to quantify the association between post-operative complications, a major cost driver, and the cost of investigations following total hip or knee arthroplasty.

**Methods:**

This is a prospective cohort study of consecutive patients undergoing primary total hip or knee arthroplasty at an Australian public hospital. We measured the number and cost of imaging and pathology tests performed during the acute hospital stay and used linear regression to quantify the association between complication status and investigation costs.

**Results:**

Five hundred patients were included in the analysis. On average, those with complications received more tests, and more expensive tests. The mean combined cost of imaging and pathology tests in patients with no complications was AU$ 187 (SD: 12.0). In comparison, patients with minor complications had a mean additional cost of AU$ 270 (SD: 31.0), and those with major complications had a mean additional cost of AU$ 493 (SD: 54.2) (*p* < 0.001).

**Conclusions:**

In patients undergoing hip or knee arthroplasty, investigation costs are substantially greater in the presence of either minor or major complications. With growing volumes of total hip and total knee arthroplasties, a potential focus of future research could include optimising investigation practices for patients with and without complications.

## Background

Total hip and total knee arthroplasty (THA/TKA) are among the most common types of surgeries performed in Australia, where over 100,000 arthroplasties are performed every year, mostly for the treatment of severe osteoarthritis [1]. The current cost of THA/TKA is AU$ 2 billion annually, though with an ageing population and increasing rate of obesity, the annual cost is projected to reach AU$ 5.32 billion by 2030 [2]. The increasing financial burden of arthroplasty on the health system has led to a growing interest in improving the cost efficiency and safety of these surgeries.

While there has been much research on many aspects of arthroplasty care, little has been written on the burden of post-operative investigations following either surgery. Post-operative investigations, which are used by clinicians to detect complications, can impose substantial burden both financially on the health system and physically on the patient [3]. Recent studies have questioned investigation practices following total hip or total knee arthroplasty, and they have identified existing cost-inefficiency at individual institutions [4–11]. However, a general lack of knowledge on current practices greatly limits the applicability of such findings. This knowledge gap also limits further research aiming to improve cost-efficiency of investigation practices after THA or TKA.

Our study was designed to examine current practices and costs of post-operative investigations during the acute hospital stay following THA or TKA in a public hospital setting in Australia. The study had the following objectives:

1. To describe the frequency and type of complications during the acute hospital stay in a cohort of patients undergoing primary THA and TKA patients; and
2. To quantify the association between complication status and the number and cost of imaging and pathology tests performed during the acute hospital stay.

## Methods

### Study design and study population

This prospective cohort study is nested within a larger implementation project on TKA/THA service delivery. The aim of the implementation project was to describe current practice and identify deficiencies in delivering an arthroplasty service, and to investigate the association between mobilising early after surgery and acute length of stay at hospital. The implementation project included all patients undergoing elective primary THA or TKA at Fairfield Hospital in Sydney, Australia between August 2018 and May 2019.The current sub-study was designed to describe current investigation practices and quantify the association between complications and the number and cost of imaging and pathology investigations performed during the acute post-operative period following THA and TKA. The cost of investigations is taken from the perspective of the Australian public health system. There were no further exclusion criteria for inclusion in the acute-care analysis. Patients provided informed consent to an investigator to have their data reviewed by research personnel.

### Data sources

For the implementation study, research personnel collected patient demographic, anthropometric, comorbid and procedure information. These data were collected directly from the patient during their pre-admission visit and from the hospital medical record.

Using unique patient identifiers including Medical Record Number (MRN), which is assigned by a hospital or facility, and surgery date, we extracted imaging and pathology data for each hospital admission from the electronic medical record. The imaging data contains information on each imaging test (also called procedure), including the unique procedure identifier, procedure name, time, unique patient identifier and the associated Medicare Benefits Schedule (MBS) code(s), which identify the medical services subsidised by the Australian government including associated fees [12]. The pathology data contains the same information without the associated MBS codes. We obtained cost information for pathology tests by matching each test from the record to descriptions in the 2019 MBS Book [12]. When descriptions in the extracted data did not accurately match those listed in the MBS, we consulted the department managers of radiology and pathology, as well as the clinical nurse consultant to ensure valid cost estimations. We costed all imaging and pathology tests at 100% of the MBS schedule fees (see Additional files 1 and 2).

### Exposure

The exposure variable was the presence of a complication. A complication was defined as any medical, physical or surgical deviation from the normal post-operative course, including adverse events [13, 14]. Prior to analyses, major and minor complications were classified as “major” or “minor” based on the invasiveness of intervention required to treat the complication, whether it resulted in a change of functional status, and whether it usually prolongs hospitalisation [13, 14] (see Additional File 3 for the classification of complications).

The study population was categorised into three groups based on complication status, which describes the presence and/or severity of complications experienced during the acute hospital stay. The first group experienced no complications during their stay; the second group experienced minor complications only; and the third group experienced at least one major complication, with or without minor complications.

### Outcome

The primary outcome was the cost of imaging and pathology tests received by each patient, by exposure category. Secondary outcomes included the number and type of tests. The timeframe of measurement was the acute post-operative period, starting on the day of surgery and ending when the patient was discharged from hospital or discharged from the surgical ward to in-hospital rehabilitation.

### Data analysis

For descriptive statistics, we used one-way analysis of variance (ANOVA) to compare continuous variables and chi-squared tests to compare categorical variables by group. After assessing distributional assumptions, we used negative binomial models to estimate the relationship between complication status and the number of tests, as the number of tests received during a stay followed a count distribution with overdispersion [15]. We used linear regression models to estimate costs by exposure group. Although the distribution of cost was skewed, we chose to use a linear model without transformation in order to provide an informative measure of the total cost for the health system [16].

We risk-adjusted all models for known and suspected confounders, including age, sex, body mass index (BMI), procedure, operation time and co-morbidities, including anxiety and depression, cancer (past and current), diabetes mellitus, dementia, hypertension, hyperlipidaemia, hyperthyroidism, osteoporosis, urinary incontinence, chronic urinary tract infection, and autoimmune, cardiac, chronic respiratory, cerebrovascular, central nervous system, liver, renal, gastro-oesophageal reflux disease, and past venous thromboembolism. We used R Version 3.6.0 (www.r-project.org) to conduct all analyses [17].

## Results

### Study cohort

The project recruited 521 consecutive patients who underwent elective primary THA or TKA at Fairfield Hospital, Sydney between August 2018 and May 2019. Out of 521 patients recruited, 500 were included in our study. Of the 21 patients excluded, 19 had imaging and pathology records that were either incomplete or inaccessible to our investigators, one patient died intra-operatively, and one patient had two admissions during the study period, of which the second admission was excluded.

### Characteristics of the study population

The mean age of our cohort was 67.9 years (SD: 9.6) and 329 (65.8%) were female. Three hundred and sixty-nine patients (73.8%) received a TKA, whereas 131 (26.2%) received a THA. Most patients (405; 81.0%) experienced no complications during their acute hospital stay; 73 (14.6%) experienced only minor complications and 22 (4.4%) experienced at least one major complication. Of the 22 who experienced a major complication, 6 (27.3%) also experienced minor complications. The three groups were similar at baseline, though a greater proportion of people with complications also had cardiovascular disease (*p* = 0.04), hypertension (*p* = 0.04) and renal impairment (*p* = 0.04). The median length of stay was 4.0 days (range: 1.0–13.0) in patients with no complications, 6.0 days (range: 2.0–21.0) in those who experienced minor complications and 5.0 days (range: 2.0–14.0) in those with major complications (*p* < 0.001). ﻿The rate of admission to an intensive care unit or high-dependency unit (ICU/HDU) was 3.2% in patients with no complications, 9.6% in patients with minor complications, and 18.2% in those with major complications (*p* = 0.001; Table 1).

**Table 1:** Demographic, anthropometrics and comorbidity profiles of the cohort by complication status

	No complications *N* = 405	Minor only *N* = 73	Major, at least one *N* = 22	*p*-value
Demographic characteristics				
Female	271 (66.9%)	46 (63.0%)	12 (54.5%)	0.425
Mean age, years (SD)	67.8 (9.4)	69.2 (9.7)	65.5 (11.9)	0.258
Primary Diagnosis:				0.289
Osteoarthritis	390 (96.3%)	70 (95.9%)	20 (90.9%)	
Other	15 (3.7%)	3 (4.1%)	2 (9.1%)	
BMI (kg/m^2^):				.
< 18	0 (0.0%)	1 (1.4%)	0 (0.0%)	
≥ 18 < 25	42 (10.4%)	9 (12.3%)	2 (9.1%)	
≥ 25 < 30	97 (24.0%)	17 (23.3%)	4 (18.2%)	
≥ 30 < 35	134 (33.1%)	19 (26.0%)	8 (36.4%)	
≥ 35	132 (32.6%)	27 (37.0%)	8 (36.4%)	
Smoking:				0.725
Never smoked	305 (75.3%)	55 (75.3%)	14 (63.6%)	
Past smoker	63 (15.6%)	11 (15.1%)	5 (22.7%)	
Current smoker	37 (9.1%)	7 (9.6%)	3 (13.6%)	
Co-morbidities				
Anxiety or depression	66 (16.3%)	11 (15.1%)	3 (13.6%)	0.968
Autoimmune disease	11 (2.7%)	0 (0.0%)	2 (9.1%)	0.064
Cardiovascular	93 (23.0%)	27 (37.0%)	6 (27.3%)	0.039
Chronic respiratory	78 (19.3%)	13 (17.8%)	3 (13.6%)	0.868
Cancer (current or past)	52 (12.8%)	8 (11.0%)	2 (9.1%)	0.924
Cerebrovascular	26 (6.4%)	7 (9.6%)	4 (18.2%)	0.078
Central nervous system (other)	10 (2.5%)	2 (2.7%)	1 (4.5%)	0.576
Diabetes mellitus	86 (21.2%)	22 (30.1%)	7 (31.8%)	0.151
Dementia	2 (0.5%)	2 (2.7%)	0 (0.0%)	0.165
Gastro-intestinal or liver disease	142 (35.1%)	29 (39.7%)	6 (27.3%)	0.534
Hypertension	271 (66.9%)	59 (80.8%)	13 (59.1%)	0.038
Hyperlipidaemia	165 (40.7%)	30 (41.1%)	7 (31.8%)	0.702
Renal impairment	21 (5.2%)	8 (11.0%)	3 (13.6%)	0.041
Hypothyroidism	29 (7.2%)	5 (6.8%)	2 (9.1%)	0.821
Venous thromboembolism (past)	19 (4.7%)	0 (0.0%)	0 (0.0%)	0.125
Osteoporosis	17 (4.2%)	5 (6.8%)	0 (0.0%)	0.514
Urinary incontinence	21 (5.2%)	4 (5.5%)	2 (9.1%)	0.551
Urinary tract infection (chronic)	6 (1.5%)	1 (1.4%)	0 (0.0%)	1.000
ASA Classification:				0.370
1	21 (5.2%)	2 (2.8%)	1 (4.5%)	
2	201 (49.9%)	28 (38.9%)	9 (40.9%)	
3	180 (44.7%)	42 (58.3%)	12 (54.5%)	
4	1 (0.2%)	0 (0.0%)	0 (0.0%)	
Missing	2 (0.5%)	1 (1.4%)	0 (0.0%)	
Procedure:				0.025
Total hip	99 (24.4%)	21 (28.8%)	11 (50.0%)	
Total knee	306 (75.6%)	52 (71.2%)	11 (50.0%)	
Number of joints:				0.049
Unilateral	11 (2.7%)	5 (6.8%)	2 (9.1%)	
Bilateral	394 (97.3%)	68 (93.2%)	20 (90.9%)	
Operation time, minutes (SD)	102.0 (24.7)	110.7 (32.5)	108.0 (30.1)	0.026
Median length of Stay, days [range]	4.0 [1.0;13.0]	6.0 [2.0;21.0]	5.0 [2.0;14.0]	< 0.001
Length of Stay:				< 0.001
≤ 3 days	142 (35.1%)	10 (13.7%)	4 (18.2%)	
4 to 6	219 (54.1%)	37 (50.7%)	11 (50.0%)	
7 or more	44 (10.9%)	26 (35.6%)	7 (31.8%)	
HDU/ICU Admission	13 (3.2%)	7 (9.6%)	4 (18.2%)	0.001

*Abbreviations*: *SD* standard deviation, *ASA* American Society of Anesthesiologists, *BMI* body mass index, *HDU* high-dependency unit, *ICU* intensive care unit

### Complications

We recorded 116 complications in 95 people, 94 of which were minor complications, and 22 of which were major complications. A greater proportion of patients undergoing THA experienced major complications compared to those undergoing TKA (11 out of 131 [8.4%] of THA patients versus 11 out of 369 [3.0%] of TKA patients; *p* = 0.025) (Additional File 4). Joint-related complications affected 50 (10.0%) patients during their stay. The most common joint-related complication was minor wound bleeding or oozing requiring vacuum dressing, which affected 37 (7.4%) patients. Other types of joint-related complications were rare, such as fracture (*n* = 4; 0.8%), major bleeding (*n* = 3; 0.6%) and superficial surgical site infection (*n* = 3; 0.6%). On the other hand, non-joint related complications affected 56 (11.2%) patients during their stay. The most common minor non-joint-related complications was cardiac arrhythmia (*n* = 11; 2.2%), followed by delirium (*n* = 10; 2.0%), urinary tract infection (*n* = 8; 1.6%) and electrolyte disturbance (*n* = 7; 1.4%), and the most common major non-joint-related complication was a respiratory complication (*n* = 7; 1.4%). No venous thromboembolisms were diagnosed during the acute care period in this cohort though several patients were investigated for this outcome. For all complications recorded see Additional File 4.

### Cost of investigations

#### Imaging and pathology cost per patient by complication status

﻿Complication status was associated with both the total number and total cost of imaging and pathology tests per patient. ﻿The mean number of imaging tests was 1.5 (SD: 0.7) in patients with no complications, 2.4 (SD: 1.6) in patients with only minor complications and 3.5 (SD: 2.3) in those with at least one major complication (*p* < 0.001). The mean number of pathology tests was 5.5 (SD: 4.3) in patients with no complications, 13.5 (SD: 14.9) in patients with only minor complications and 18.3 (SD: 18.0) in those with at least one major complication (*p* < 0.001). In an adjusted negative binomial model, we found that patients with only minor complications received more imaging tests (aIRR: 1.49; 95% CI:1.24–1.78) and more than double the number of pathology tests (aIRR: 2.07; 95% CI:1.74–2.47) relative to those with no complications. Patients with at least one major complication received more than double the number of imaging (aIRR: 2.07; 95% CI:1.59–2.65) and triple the number of pathology tests (aIRR: 3.00; 95% CI:2.26–4.04) compared to patients with no complications.

**Fig. 1:**
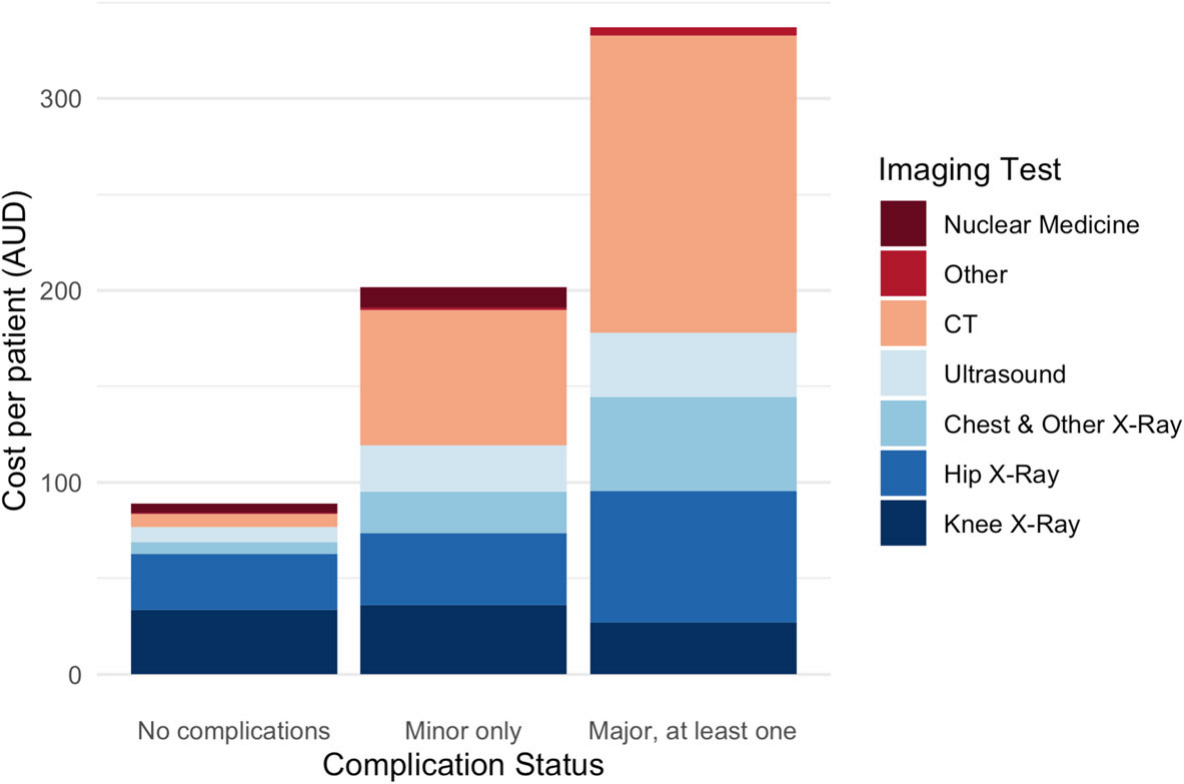
Mean cost of imaging tests performed per patient, by complication status

**Fig. 2:**
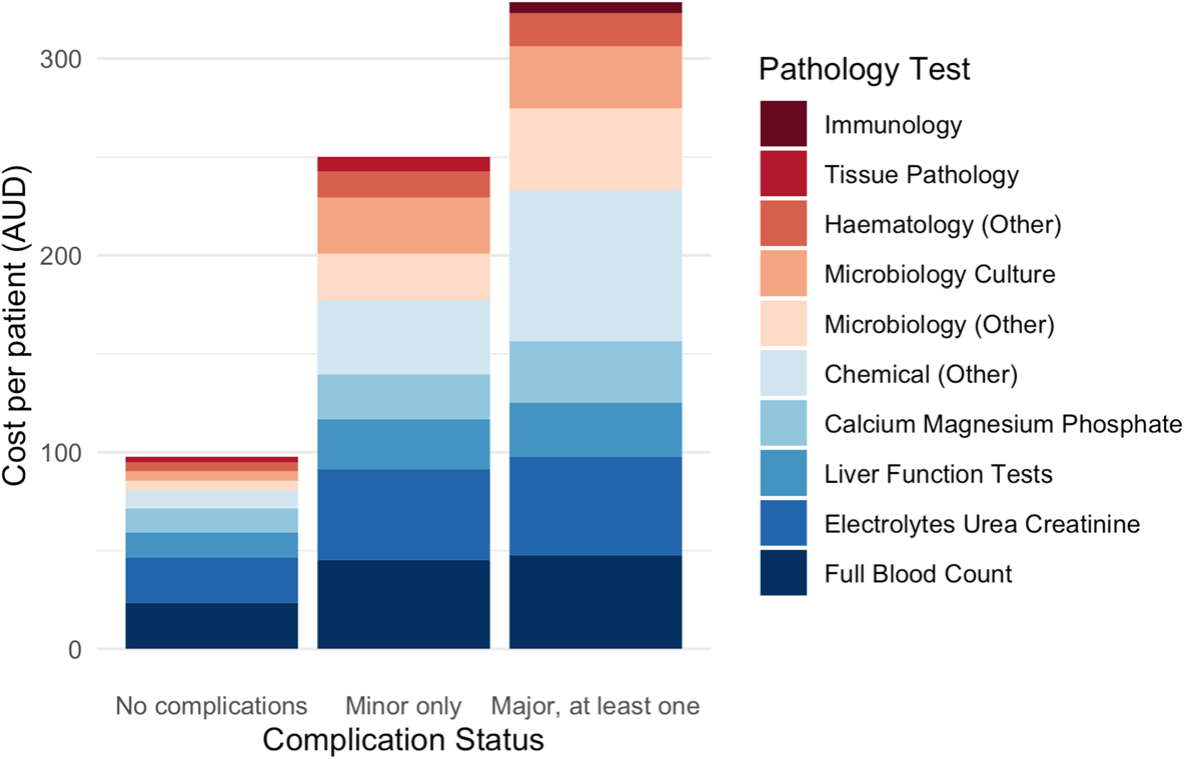
Mean cost of pathology tests performed per patient, by complication status

﻿As the number of tests increased with each level of complication status, the mean total cost increased concomitantly (Table 2; Figs. 1 & 2). In patients with no complications, the total imaging costs were AU$ 88.9 (SD: 101.8) and pathology costs were AU$ 97.7 (SD: 83.6) per patient. ﻿In an adjusted linear regression model, patients with only minor complications incurred more than double the cost compared with patients with no complications, with mean additional costs per patient of AU$ 103.7 (95% CI: 66.2–141.1) for imaging and AU$ 136.3 (95% CI: 98.0–174.5) for pathology. Among patients with at least one major complication, the costs for imaging and pathology tests were more than three times higher than for those with no complications, with mean additional costs per patient of AU$ 231.4 (95% CI: 167.4–295.5) for imaging and AU$ 217.2 (95% CI: 151.8–282.6) for pathology.

**Table 2:** Mean cost (in AU$) of imaging and pathology tests performed per patient, with additional cost by complication status in AU$

	No complications *N* = 405	Minor only *N* = 73	Major, at least one *N* = 22
**Imaging**			
Mean cost (SD)	88.9 (101.8)	201.7 (251.3)	337.4 (299.4)
Additional cost^a^, unadjusted (95% CI)	Reference	+ 112.8 (76.3–149.4)	+ 248.5 (185.7–311.4)
Additional cost, adjusted^b^ (95% CI)	Reference	+ 103.7 (66.2–141.1)	+ 231.4 (167.4–295.5)
**Pathology**			
Mean cost (SD)	97.7 (83.6)	250.3 (285.5)	329.0 (323.8)
Additional cost^a^, unadjusted (95% CI)	Reference	+ 152.7 (115.7–189.6)	+ 231.3 (167.6–295.0)
Additional cost, adjusted^b^ 95% CI)	Reference	+ 136.3 (98.0–174.5)	+ 217.2 (151.8–282.6)

^a^ Additional cost represents the cost that is added when the specified type of complication is present, compared to when it is absent

^b^ Adjusted for age, sex, procedure, ASA score, operation time (minutes), anxiety and depression, cancer (past and current), diabetes mellitus, dementia, hypertension, hyperlipidaemia, hyperthyroidism, osteoporosis, urinary incontinence, chronic urinary tract infection, and autoimmune, cardiac, chronic respiratory, cerebrovascular, central nervous system, liver, renal, gastro-oesophageal reflux disease, and past venous thromboembolism

Regardless of complication status, every ICU/HDU admission prompts a series of pathology screening tests used to detect multi-drug resistant microorganisms. The mean number of pathology tests in those admitted to ICU/HDU was 26.2 (SD: 18.0), compared to 6.3 (SD: 6.6) in those who were not admitted to ICU/HDU (*p* < 0.001).

#### Imaging and pathology tests by overall cost contribution

**Fig. 3:**
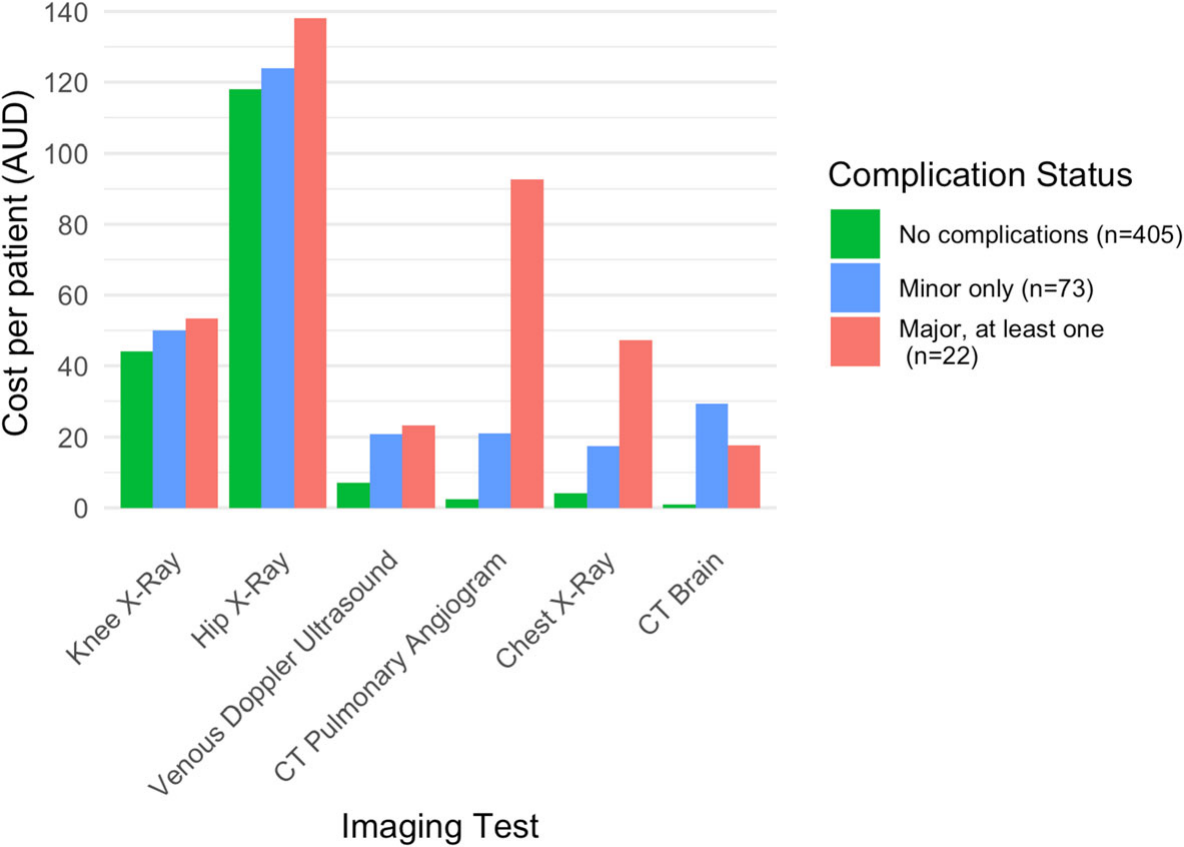
Largest contributors of imaging cost: cost per patient by complication status

The imaging tests contributing most to the overall cost burden of this cohort were: hip x-ray (mean AU$ 121.0 per THA patient; SD = 31.1; *n* = 277), knee x-ray (mean AU$45.2 per TKA patient; SD = 9.5; *n* = 384), venous doppler ultrasound (mean AU$ 9.8 per patient; SD = 41.1; *n* = 29), CT pulmonary angiogram (mean AU$ 9.2 per patient; SD = 67.9; *n* = 9), chest x-ray (mean AU$ 8.0 per patient; SD = 24.3; *n* = 85) and CT brain (mean AU$ 5.9 per patient; SD = 37.6; *n* = 15) (Additional File 5). Of these imaging tests, only hip/knee x-rays were performed routinely in all patients (1.0 knee x-ray per TKA patient; 2.1 hip x-rays per THA patient). Other imaging tests were performed selectively, and their use was often associated with complication status (*p* < 0.001) (Fig. 3; see Additional File 6-7 for the mean number and cost of these tests by complication status).

**Fig. 4:**
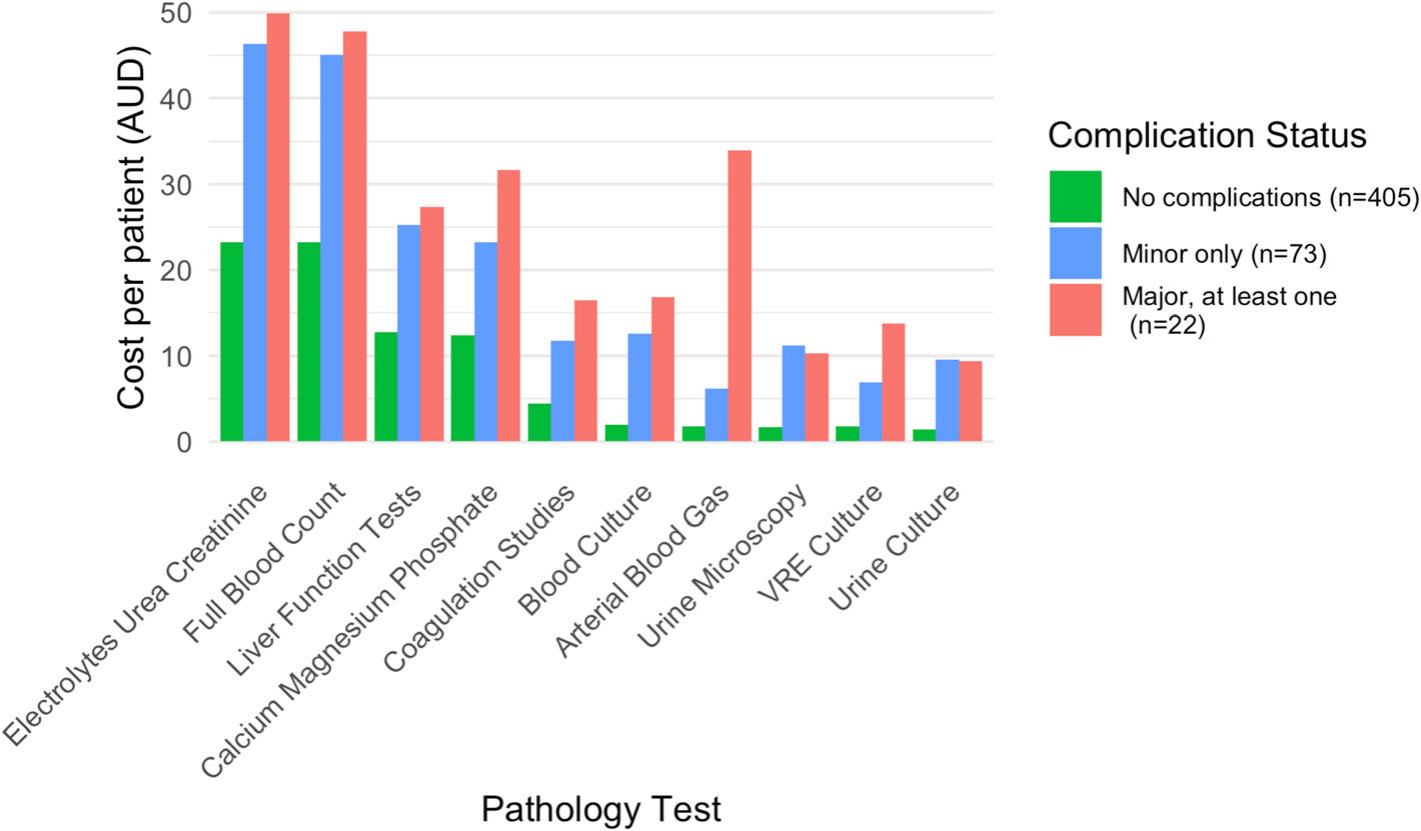
Largest contributors of pathology cost: cost per patient by complication status

The pathology tests contributing most to overall cost burden were: electrolytes/urea/creatinine (mean: AU$27.8 per patient), full blood count (mean AU$27.5), liver function tests (mean AU$15.2), calcium/magnesium/phosphate (mean AU$14.8), coagulation studies (mean AU$6), blood culture (mean AU$4.2), arterial blood gas (mean AU$3.9), urine microscopy (mean AU$3.5), vancomycin-resistant enterococci (VRE) culture (mean AU$3.0) and urine culture (mean AU$3.0) (Additional File 5). Each patient received at least one set of blood tests including electrolytes/urea/creatinine, full blood count and calcium/magnesium/phosphate. The use and cost per patient of all pathology tests increased significantly with the presence and severity of complications (*p* < 0.001; Fig. 4).

## Discussion

### Summary of main findings

We found that the cost of investigations was a consistent contributor to the cost of the acute hospital stay, and that these costs increased significantly with the presence and severity of complications. Five out of six of the largest contributors to the total cost burden incurred by the cohort were tests performed routinely in all patients: hip/knee x-ray for imaging, and full blood count, electrolytes/urea/creatinine and calcium/magnesium/phosphate for pathology. Of this list, the pathology investigations were often ordered repeatedly in those with complications, which may be interpreted as a necessary component of care during a prolonged hospital stay.

### Interpretation of main findings

Our study results align with previously reported findings that patients with complications incur a 35–50% higher overall cost during the acute stay compared to their counterparts with no complications (AU$20,241 vs. 28,249 for THA and AU$19,432 vs. 26,729 for TKA) [18]. Patients with complications also have longer hospital stays [19–21] and are more likely to be admitted to HDU/ICU [3, 22] than those without. In fact, a single HDU/ICU admission automatically prompts a set of more than 20 routine screening tests for multi-drug resistant organisms at our institution. Though the presence of the association has previously been inferred, this study was the first to quantify and compare the levels of cost between complication groups.

Specifically, despite the use of different definitions to capture complications and case-mix complexity, the cost estimates reported in this study are similar to figures previously published by the Independent Hospital Pricing Authority (IHPA) [18]. Firstly, the IHPA reported a more than three-fold difference between “minor complexity” cases and “major complexity” cases (AU$ 210 vs. 799 for TKA and AU$ 281 vs. 1034 for THA). The IHPA “minor complexity” cases are roughly equivalent to those of our “no complications” and “minor complications” groups; while the costs of the IHPA “major complexity” cases are higher than those of our “major complications” group. Secondly, the cost difference between THA and TKA patients seen in the IHPA figures was also observed in our study cohort, reflecting higher rates of complications and more routine imaging tests in THA patients. Thus, our findings are within an expected range, both in terms of the absolute cost estimates and the relative differences between complication groups and THA/TKA groups. Building upon these IHPA figures, our study also quantifies investigations costs for patients with no complications, highlighting the consistent contribution of routine investigations to the overall cost burden.

### Clinical relevance

The cost of post-operative investigations contributes consistently to the cost of the acute hospital stay, and the absolute financial burden of investigations will only increase with the growing volume of THA and TKA surgeries. The annual volume of total hip and knee arthroplasty in Australia amounts to over 120,000 procedures at a total cost of AU$ 2 billion [1, 2]. Given the high volume, optimising investigation approaches may provide substantial cost savings even when the percentage of overall cost reduction is small. Recent studies have identified examples of inefficient routine investigations, including radiographs and blood tests, during post-arthroplasty care in tertiary centres in USA and in China [5–11]. In Australia, only one similar study has been conducted; it found that routine post-operative radiographs did not alter inpatient management of any patients, despite 23% having atypical radiographic findings in that cohort [4]. It is evident that further work is required to evaluate the cost-effectiveness of post-operative investigations in Australia. In this light, our study could act as a foundational piece of research by quantifying the current burden of post-operative investigations following THA or TKA in patients with and without complications.

### Strengths and limitations

To the best of our knowledge, this is the first study to quantify the cost of investigations during the acute hospital stay following total hip or knee arthroplasty by complication status. The accuracy of complication data was enhanced by the prospective study design, where purposely collected clinical data were used to determine complication status of patients. In addition, although the absolute cost of investigations may vary between countries or even institutions within the same jurisdiction, the relative cost increase observed in this study is likely generalisable to other THA or TKA cohorts in the Australian public health systems, as our study population included all patients admitted to a public high-volume joint replacement centre within the predetermined timeframe.

A limitation of our study is that it provides limited insight into the clinical utility of the investigations. As we did not have information about the indications for investigations, we are unable to comment on the cost-efficiency or appropriateness of investigations practices in this cohort. Second, our analysis accounts for only the direct costs from the perspective of the Australian public health system. We did not consider indirect costs to the hospital such as general administrative costs or the maintenance of health records, nor did we address indirect costs to the patient such as those related to missed work or decreased work productivity. Thus, although the direct costs alone amount to a substantial burden given the high volume of these procedures, the total cost to the health system and the patient extends far beyond the costs measured in this study. Finally, we used manually matched Medicare Benefits Schedule cost items for pathology tests. Although we fully adhered to test descriptions in the Medicare Benefits Schedule Book and consulted with the department manager of pathology for validation, this manual method is inferior to using automatically-generated billing data.

## Conclusion

In this prospective cohort study of 500 patients undergoing total hip or knee arthroplasty, post-operative complications, depending on severity, were associated with a roughly two-to-three-fold increase in investigation costs during the acute hospital stay. With growing volumes of total hip and total knee arthroplasties, the focus of future research should be placed on optimising investigation practices to reduce both financial and physical burden for both patients with and without complications.

## Supplementary Information

**Additional file 1.** Pathology costs. Pathology test items with Medicare Benefits Schedule codes and fees.

**Additional file 2.** Imaging costs. Imaging test items with Medicare Benefits Schedule codes and fees.

**Additional file 3.** Classification of complications. Classification of complications into: major and minor; joint-related and non-joint-related complications.

**Additional file 4.** Complications in study population. Number and type of complications recorded in study population overall and by joint.

**Additional file 5.** Cost and number of tests performed. Relationship between cost and number of tests performed (per 100 patients).

**Additional file 6.** Mean number of tests by complication status. Mean number (SD) of imaging and pathology tests per 100 patients by complication status.

**Additional file 7.** Mean cost of tests by complications status. Mean cost (SD) of imaging and pathology tests per patient by complication status in AU$.

## Data Availability

No additional data are available.

## Abbreviations

ASA: American Society of Anesthesiologists
BMI: Body mass index
CI: Confidence interval
CT: Computed tomography
HDU: High dependency unit
ICU: Intensive care unit
IHPA: Independent Hospital Pricing Authority
IRR: Incidence rate ratio
SD: Standard deviation
THA: Total hip arthroplasty
TKA: Total knee arthroplasty
VRE: Vancomycin-resistant enterococci
