# Supplementary material for: Cost of investigations during the acute hospital stay following total hip or knee arthroplasty, by complication status
Source: BMC Health Serv Res. 2020 Nov 12;20:1036. doi: 10.1186/s12913-020-05892-1 (PMC7659097; doi:10.1186/s12913-020-05892-1)
Supplement: Supplementary file 1 — Additional file 1. Pathology costs. Pathology test items with Medicare Benefits Schedule codes and fees. [file 12913_2020_5892_MOESM1_ESM.docx]

Pathology test items with Medicare Benefits Schedule codes and fees

| **Pathology test item** | **MBS**  **Code 1** | **MBS Fee 1**  **(AUD)** | **MBS**  **Code 2** | **MBS Fee 2**  **(AUD)** | **Comment** |
| --- | --- | --- | --- | --- | --- |
| Active B12 (Holotranscobalamin) Level | 66839 | 42.95 |  |  |  |
| Add on Lab Test(s) |  |  |  |  | Non-cost item |
| Adrenocorticotropic Hormone Level | 66695 | 30.5 |  |  |  |
| Albumin | 66500 | 9.7 |  |  |  |
| Alpha 1 Antitrypsin Analysis | 66635 | 20.1 |  |  |  |
| Amylase and Lipase | 66503 | 11.65 |  |  |  |
| Antineutrophil Cytoplasmic Antibodies | 71153 | 34.55 |  |  |  |
| Antinuclear Antibodies | 71097 | 24.45 |  |  |  |
| AP Histopathology Report |  |  |  |  | Non-cost item |
| Apixaban Level | 65147 | 37.9 |  |  |  |
| APTT | 65120 | 13.7 |  |  |  |
| Arterial Blood Gas & Electrolytes & Lactate Analysis | 66566 | 33.7 |  |  |  |
| Blood Culture  1 test within same stay  2  3  4 | 69354  69357  69360  69360 | 30.75  61.45  92.2  92.2 | 69354 | 30.75 |  |
| Blood Group | 65090 | 22 |  |  |  |
| Blood Group Ab Screen and Crossmatch | 65099 | 108.9 |  |  |  |
| Blood Group and Antibody Screen | 65096 | 41 |  |  |  |
| C Reactive Protein | 66500 | 9.7 |  |  |  |
| Calcium Magnesium Phosphate | 66506 | 13.65 |  |  |  |
| Cancellation Coagulation |  |  |  |  | Non-cost item |
| Cancellation Haematology |  |  |  |  | Non-cost item |
| Chemistry Comment |  |  |  |  | Non-cost item |
| Cholesterol | 66500 | 9.7 |  |  |  |
| Clearance Screen MRSA | 69303 | 22 |  |  |  |
| Coagulation (INR & APTT) | 65123 | 20.35 |  |  |  |
| Coagulation Comment |  |  |  |  | Non-cost item |
| Cortisol Level | 66695 | 30.5 |  |  |  |
| Creatine Kinase | 66500 | 9.7 |  |  |  |
| Culture Eye | 69303 | 22 |  |  |  |
| Culture Genital | 69306 | 33.75 |  |  |  |
| Culture Mycobacteria  1 specimen per episode of care  2 | 69324  69327 | 43  85 |  |  |  |
| Culture Nose | 69303 | 22 |  |  |  |
| Culture Respiratory | 69318 | 33.75 |  |  |  |
| Culture Tip | 69306 | 33.75 |  |  |  |
| Culture Tissue | 69321 | 48.15 |  |  |  |
| Culture Urine | 69333 | 20.55 |  |  |  |
| Culture Wound Operative | 69321 | 48.15 |  |  |  |
| Culture Wound Superficial Lesion | 69306 | 33.75 |  |  |  |
| Cut Slide IH R/Cut |  |  |  |  | Non-cost item; related to tissue pathology specimen |
| Cut Slide SS R/Cut |  |  |  |  | Non-cost item |
| Cytomegalovirus Immune Status Serology | 69384 | 15.65 |  |  |  |
| D-Dimer Assay | 65120 | 13.7 |  |  |  |
| Decal Specimen (L,C) |  |  |  |  | Non-cost item |
| Digoxin Level | 66800 | 18.15 |  |  |  |
| Direct Antiglobulin Testing | 65114 | 9.1 |  |  |  |
| Electrolytes Liver Function Full Blood Count | 66512 | 17.7 |  |  |  |
| Electrolytes Urea Creatinine | 66512 | 17.7 |  |  |  |
| Electrolytes Urea Creatinine Glucose | 66512 | 17.7 |  |  |  |
| Embed Blk Cut H&E |  |  |  |  | Non-cost item; related to tissue pathology specimen |
| Epstein Barr virus Serology | 69472 | 15.65 |  |  |  |
| Erythrocyte Sedimentation Rate | 65060 | 7.85 |  |  |  |
| Faeces Bacterial Screen | 69345 | 52.9 |  |  |  |
| Faeces Bacterial Screen. | 69345 | 52.9 |  |  |  |
| Faeces Clost.difficile Toxin DNA Detection | 69363 | 28.65 |  |  |  |
| Faeces Microscopy |  |  |  |  | Non-cost item |
| Fibrinogen | 65120 | 13.7 |  |  |  |
| Full Blood Count | 65070 | 16.95 |  |  |  |
| Full Blood Count and ESR | 66512 | 17.7 |  |  |  |
| Glomerular Basement Membrane Antibodies. | 71153 | 34.55 |  |  |  |
| Glucose Fasting | 66500 | 9.7 |  |  |  |
| Glucose Random | 66500 | 9.7 |  |  |  |
| Glycosylated HbA1c ?Diabetic | 66551 | 16.8 |  |  |  |
| Glycosylated HbA1c Known Diabetic | 66841 | 16.8 |  |  |  |
| Haptoglobin | 66632 | 20.1 |  |  |  |
| Hepatitis A Abs Acute Infection  1 test per episode  2 tests | 69475  69478 | 15.65  29.25 |  |  |  |
| Hepatitis B Core Antibodies Total | *As above* |  |  |  |  |
| Hepatitis B Serology | *As above* |  |  |  |  |
| Hepatitis B Surface Antibodies | *As above* |  |  |  |  |
| Hepatitis B Surface Antigens | *As above* |  |  |  |  |
| Hepatitis C Antibodies | *As above* |  |  |  |  |
| ICU Admission MRSA Screen |  |  |  |  | Non-cost item  (Inoqula MRSA culture) |
| ICU Admission Resistant GNR Screen |  |  |  |  | Non-cost item  (Inoqula MRO culture) |
| ICU Admission VRE Screen |  |  |  |  | Non-cost item  (Inoqula VRE Screen) |
| ICU Discharge MRSA Screen |  |  |  |  | Non-cost item  (Inoqula MRSA culture) |
| ICU Discharge Resistant GNR Screen |  |  |  |  | Non-cost item  (Inoqula MRO culture) |
| ICU Discharge VRE Screen |  |  |  |  | Non-cost item  (Inoqula VRE Screen) |
| ICU FRF Infection Ctl Admission Screen |  |  |  |  | Non-cost item  (administrative prompt) |
| ICU FRF Infection Ctl Discharge Screen |  |  |  |  | Non-cost item  (administrative prompt) |
| IH MNF116 | 72846 | 59.6 |  |  |  |
| IH Negative |  |  |  |  | Non-cost item |
| Immunoglobulin E Level | 71075 | 23 |  |  |  |
| Inoqula MRO culture | 69303 | 22 |  |  |  |
| Inoqula MRSA culture | 69303 | 22 |  |  |  |
| Inoqula VRE culture | 69312 | 33.75 |  |  |  |
| INR | 65120 | 13.7 |  |  |  |
| Iron Studies | 66596 | 32.55 |  |  |  |
| Lactate Analysis | 66500 | 9.7 |  |  |  |
| Lactate Dehydrogenase | 66500 | 9.7 |  |  |  |
| Lipase Analysis | 66500 | 9.7 |  |  |  |
| Lipids | 66503 | 11.65 |  |  |  |
| Liver Function Tests | 66512 | 17.7 |  |  |  |
| Low Density Lipoprotein Cholesterol | 66539 | 30.6 |  |  |  |
| Neutrophil Cytoplasmic Antibodies WZ | 71153 | 34.55 |  |  |  |
| Osmolality | 66563 | 24.7 |  |  |  |
| Parathyroid Hormone Level | 66695 | 30.5 |  |  |  |
| POC GEM BG  1 test within same day  2 tests  3  4 | 66566  66569  66572  66575 | 33.7  42.6  51.55  60.45 |  |  | Arterial Blood Gas |
| POC INR | 65120 | 13.7 |  |  |  |
| Pre Admission MRSA Screen | 69303 | 22 |  |  | Non-cost item |
| Recut H&E |  |  |  |  |  |
| Respiratory Virus RNA Detection. |  |  |  |  |  |
| Reticulocytes | 65072 | 10.2 |  |  |  |
| Rivaroxaban Level | 65147 | 37.9 |  |  |  |
| Screen for MRSA | 69303 | 22 |  |  |  |
| Ser Folate | 66840 | 23.6 |  |  |  |
| SS MZN ( L ) |  |  |  |  | Non-cost item |
| SS PAS |  |  |  |  | Non-cost item |
| SS Perls |  |  |  |  | Non-cost item; related to tissue pathology specimen |
| Thyroid Function Tests Analysis | 66719 | 34.8 |  |  |  |
| Thyroid Stimulating Hormone | 66716 | 25.05 |  |  |  |
| Thyroxine Free | 66719 | 34.8 |  |  |  |
| Tissue Histopathology Request |  |  |  |  | Non-cost item |
| Triglycerides | 66500 | 9.7 |  |  |  |
| Triiodothyronine Free Analysis | 66719 | 34.8 |  |  |  |
| Troponin T - high sensitive  1 test within 24 hours  2 or more | 66518  66519 | 20.05  40.15 |  |  |  |
| Tryptase | 71198 | 40.55 |  |  |  |
| Urine Legionella pneumophila Ags | 69384 | 15.65 |  |  |  |
| Urine Microscopy. | 69333 | 20.55 |  |  |  |
| Urine Osmolality Random | 66563 | 24.7 |  |  |  |
| Urine Pneumococcus Antigens | 69384 | 15.65 |  |  |  |
| Urine Sodium and Osmolality Random | 66500 | 9.7 | 66563 | 24.7 |  |
| Urine Sodium Random | 66500 | 9.7 |  |  |  |
| Van Nucleic Acid Detection |  |  |  |  |  |
| Vasopressin and Osmolality | 66695 | 30.5 | 66563 | 24.7 |  |
| Vasopressin Level | 66695 | 30.5 |  |  |  |
| Vit B12 and Serum Folate | 66838 | 23.6 | 66840 | 23.6 |  |
| Vit B12, Serum Folate and Iron Studies | 66838 | 23.6 | 66840 | 23.6 |  |
| Vitamin B12 Level | 66838 | 23.6 |  |  |  |
| Vitamin D 25 Hydroxy Level | 66833 | 30.05 |  |  |  |
| WZ Urine Microscopy. | 69333 | 20.55 |  |  |  |
| X31 (72816) | 72816 | 86.35 |  |  |  |
| X41 (72823) | 72823 | 97.15 |  |  |  |
| X42-4 (72824) | 72824 | 141.35 |  |  |  |
| X5 (72830) | 72830 | 274.15 |  |  |  |
| XIP/IF1-3 (72846) | 72846 | 59.6 |  |  |  |
