# Supplementary material for: Cost of investigations during the acute hospital stay following total hip or knee arthroplasty, by complication status
Source: BMC Health Serv Res. 2020 Nov 12;20:1036. doi: 10.1186/s12913-020-05892-1 (PMC7659097; doi:10.1186/s12913-020-05892-1)
Supplement: Supplementary file 2 — Additional file 2. Imaging costs. Imaging test items with Medicare Benefits Schedule codes and fees. [file 12913_2020_5892_MOESM2_ESM.docx]

Imaging test items with Medicare Benefits Schedule codes and fees

| **Imaging test item** | **MBS Code 1** | **MBS Fee 1**  **(AUD)** | **MBS Code 2** | **MBS Fee 2**  **(AUD)** | **MBS Code 3** | **MBS Fee 3**  **(AUD)** |
| --- | --- | --- | --- | --- | --- | --- |
| CT Abdomen including Pelvis | 56507 | 480.05 |  |  |  |  |
| CT Brain | 56001 | 195.05 |  |  |  |  |
| CT Circle of Willis | 57350 | 510 |  |  |  |  |
| CT Facial Bones | 56022 | 225 |  |  |  |  |
| CT Hip Right | 56409 | 250 |  |  |  |  |
| CT Knee Right | 56619 | 220 |  |  |  |  |
| CT KUB | 56501 | 385 |  |  |  |  |
| CT Pelvis and Hip Left | 56409 | 250 |  |  |  |  |
| CT Pulmonary Angiogram | 57350 | 510 |  |  |  |  |
| CT Spine Cervical | 56220 | 240 |  |  |  |  |
| CT Spine Thoracic | 56221 | 240 |  |  |  |  |
| Fluoroscopy Biliary ERCP | 58927 | 76.45 |  |  |  |  |
| NM BMD >70 | 12323 | 102.4 |  |  |  |  |
| NM BMD Steroid | 12306 | 102.4 |  |  |  |  |
| NM Bone | 61425 | 600.7 | 61505 | 100 |  |  |
| NM Lung Ventilation and Perfusion | 61348 | 443.35 |  |  |  |  |
| NM Myocardial Perfusion Sestamibi | 11712 | 152.15 | 61307 | 834.9 | 61505 | 100 |
| OT MISC Mobile II < 1 Hour | 60506 | 63.75 |  |  |  |  |
| OT MISC Mobile II >1 Hour | 60509 | 98.9 |  |  |  |  |
| US Hips Paediatric | 55820 | 109.1 |  |  |  |  |
| US Knee Right | 55828 | 109.1 |  |  |  |  |
| US Liver | 55036 | 111.3 |  |  |  |  |
| US Lower Limb Bilateral Venous Doppler | 55244 | 169.5 |  |  |  |  |
| US Lower Limb Venous Doppler 1 Side | 55244 | 169.5 |  |  |  |  |
| US Neck | 55032 | 109.1 |  |  |  |  |
| US Renal | 55038 | 109.1 |  |  |  |  |
| US Thyroid | 55032 | 109.1 |  |  |  |  |
| XR Abdomen Supine | 58903 | 47.6 |  |  |  |  |
| XR Abdomen Supine & Erect | 58903 | 47.6 |  |  |  |  |
| XR Abdomen, Pelvis and Hips Bilateral | 57712020 | 47.15 |  |  |  |  |
| XR Ankle Left | 57521031 | 43.4 |  |  |  |  |
| XR Chest | 58503 | 47.15 |  |  |  |  |
| XR Femur and Knee Right | 57527023 | 65.75 |  |  |  |  |
| XR Foot Left | 57521030 | 43.4 |  |  |  |  |
| XR Foot Right | 57521020 | 43.4 |  |  |  |  |
| XR Hand Right | 57509020 | 39.75 |  |  |  |  |
| XR Hip Left | 57712030 | 47.15 |  |  |  |  |
| XR Hip Right | 57712020 | 47.15 |  |  |  |  |
| XR Hip Right | 57715 | 60.9 |  |  |  |  |
| XR Hips Bilateral | 57712020 | 47.15 | 57712030 | 47.15 |  |  |
| XR Hips Bilateral | 57712020 | 47.15 |  |  |  |  |
| XR Hips Bilateral | 57712030 | 47.15 |  |  |  |  |
| XR Knee Left | 57521033 | 43.4 |  |  |  |  |
| XR Knee Right | 57521023 | 43.4 |  |  |  |  |
| XR Knee Right | 57521023 | 43.4 |  |  |  |  |
| XR Knees Bilateral | 57521023 | 43.4 |  |  |  |  |
| XR Knees Bilateral | 57521033 | 43.4 |  |  |  |  |
| XR Lateral Airways | 57945 | 43.4 |  |  |  |  |
| XR Long Leg Both | 57527001 | 65.75 |  |  |  |  |
| XR Long Leg Left | 57527001 | 65.75 |  |  |  |  |
| XR Long Leg Right | 57527001 | 65.75 |  |  |  |  |
| XR Lumbar Spine | 58106 | 77 |  |  |  |  |
| XR Pelvis | 57715 | 60.9 |  |  |  |  |
| XR Pelvis and Hip Left | 57712030 | 47.15 |  |  |  |  |
| XR Pelvis and Hip Left | 57715 | 60.9 | 57712030 | 47.15 |  |  |
| XR Pelvis and Hip Left | 57715 | 60.9 |  |  |  |  |
| XR Pelvis and Hip Right | 57712020 | 47.15 |  |  |  |  |
| XR Pelvis and Hip Right | 57715 | 60.9 | 57712020 | 47.15 |  |  |
| XR Pelvis and Hip Right | 57715 | 60.9 |  |  |  |  |
| XR Pelvis Hip and Femur Right | 57715 | 60.9 | 57521024 | 43.4 | 57712020 | 47.15 |
| XR Pelvis Hip and Femur Right | 57715 | 60.9 |  |  |  |  |
| XR Spine Cervical | 58100 | 67.15 |  |  |  |  |
| XR Wrist Left | 57509031 | 39.75 |  |  |  |  |
