# Supplementary material for: Cost of investigations during the acute hospital stay following total hip or knee arthroplasty, by complication status
Source: BMC Health Serv Res. 2020 Nov 12;20:1036. doi: 10.1186/s12913-020-05892-1 (PMC7659097; doi:10.1186/s12913-020-05892-1)
Supplement: Supplementary file 3 — Additional file 3. Classification of complications. Classification of complications into: major and minor; joint-related and non-joint-related complications. [file 12913_2020_5892_MOESM3_ESM.docx]

Classification of Complications

| **Joint-related complications** |
| --- |
| ***Major*** |
| Fracture |
| Major bleeding |
| Dislocation |
| ***Minor*** |
| Wound bleeding or oozing requiring vacuum dressing |
| Superficial surgical site infection |
| Wound blister |
| **Non-joint-related complications** |
| ***Major*** |
| Death |
| Respiratory |
| Acute kidney injury |
| Myocardial infarction |
| Ketosis |
| Fall resulting in injury |
| ***Minor*** |
| Cardiac arrhythmia |
| Delirium |
| Urinary tract infection |
| Electrolyte disturbance |
| Cellulitis |
| Hypotension |
| Fall resulting in no injury |
| Fever with unknown cause |
| Anaemia |
| Atelectasis |
| Polyuria |
| ***Adverse Events*** |
| Pressure ulcer |
| Drug reaction |
