# Supplementary material for: Cost of investigations during the acute hospital stay following total hip or knee arthroplasty, by complication status
Source: BMC Health Serv Res. 2020 Nov 12;20:1036. doi: 10.1186/s12913-020-05892-1 (PMC7659097; doi:10.1186/s12913-020-05892-1)
Supplement: Supplementary file 4 — Additional file 4. Complications in study population. Number and type of complications recorded in study population overall and by joint. [file 12913_2020_5892_MOESM4_ESM.docx]

**Complications in the study population overall and by joint.**

|  | **All**  **N=500** | **THA**  **N=131** | **TKA**  **N=369** | **p-value** |
| --- | --- | --- | --- | --- |
| **Complication Status** |  |  |  | 0.025 |
| None | 405 (81.0%) | 99 (75.6%) | 306 (82.9%) |  |
| Minor only | 73 (14.6%) | 21 (16.0%) | 52 (14.1%) |  |
| Major, at least one | 22 (4.40%) | 11 (8.40%) | 11 (2.98%) |  |
| **Count of Complications** |  |  |  | 0.127 |
| 0 | 405 (81.0%) | 99 (75.6%) | 306 (82.9%) |  |
| 1 | 77 (15.4%) | 24 (18.3%) | 53 (14.4%) |  |
| 2 | 15 (3.00%) | 7 (5.34%) | 8 (2.17%) |  |
| 3 | 3 (0.60%) | 1 (0.76%) | 2 (0.54%) |  |
| **Joint-related complications** |  |  |  |  |
| ***Major*** |  |  |  |  |
| Fracture | 4 (0.80%) | 4 (3.05%) | 0 (0.00%) | 0.005 |
| Major bleeding | 3 (0.60%) | 1 (0.76%) | 2 (0.54%) | 1.000 |
| Dislocation | 1 (0.20%) | 1 (0.76%) | 0 (0.00%) | 0.262 |
| ***Minor*** |  |  |  |  |
| Wound bleeding or oozing requiring vacuum dressing | 37 (7.40%) | 17 (13.0%) | 20 (5.42%) | 0.008 |
| Superficial surgical site infection | 3 (0.60%) | 1 (0.76%) | 2 (0.54%) | 1.000 |
| Wound blister | 2 (0.40%) | 0 (0.00%) | 2 (0.54%) | 1.000 |
| **Non-joint-related complications** |  |  |  |  |
| ***Major*** |  |  |  |  |
| Respiratory | 7 (1.40%) | 3 (2.29%) | 4 (1.08%) | 0.385 |
| Acute kidney injury | 2 (0.40%) | 0 (0.00%) | 2 (0.54%) | 1.000 |
| Myocardial infarction | 1 (0.20%) | 1 (0.76%) | 0 (0.00%) | 0.262 |
| Ketosis | 1 (0.20%) | 0 (0.00%) | 1 (0.27%) | 1.000 |
| Fall resulting in injury | 1 (0.20%) | 0 (0.00%) | 1 (0.27%) | 1.000 |
| ***Minor*** |  |  |  |  |
| Cardiac arrhythmia | 11 (2.20%) | 3 (2.29%) | 8 (2.17%) | 1.000 |
| Delirium | 10 (2.00%) | 2 (1.53%) | 8 (2.17%) | 1.000 |
| Urinary tract infection | 8 (1.60%) | 5 (3.82%) | 3 (0.81%) | 0.032 |
| Electrolyte disturbance | 7 (1.40%) | 1 (0.76%) | 6 (1.63%) | 0.682 |
| Cellulitis | 5 (1.00%) | 0 (0.00%) | 5 (1.36%) | 0.333 |
| Fall resulting in no injury | 4 (0.80%) | 0 (0.00%) | 4 (1.08%) | 0.577 |
| Fever with unknown cause | 3 (0.60%) | 0 (0.00%) | 3 (0.81%) | 0.570 |
| Anaemia | 2 (0.40%) | 1 (0.76%) | 1 (0.27%) | 0.456 |
| Atelectasis | 1 (0.20%) | 0 (0.00%) | 1 (0.27%) | 1.000 |
| Polyuria | 1 (0.20%) | 0 (0.00%) | 1 (0.27%) | 1.000 |
| **Adverse Events** |  |  |  |  |
| Pressure ulcer | 3 (0.60%) | 2 (1.53%) | 1 (0.27%) | 0.169 |
| Drug reaction | 3 (0.60%) | 1 (0.76%) | 2 (0.54%) | 1.000 |
