# Supplementary figures and images for: Cost of investigations during the acute hospital stay following total hip or knee arthroplasty, by complication status
Source: BMC Health Serv Res. 2020 Nov 12;20:1036. doi: 10.1186/s12913-020-05892-1 (PMC7659097; doi:10.1186/s12913-020-05892-1)

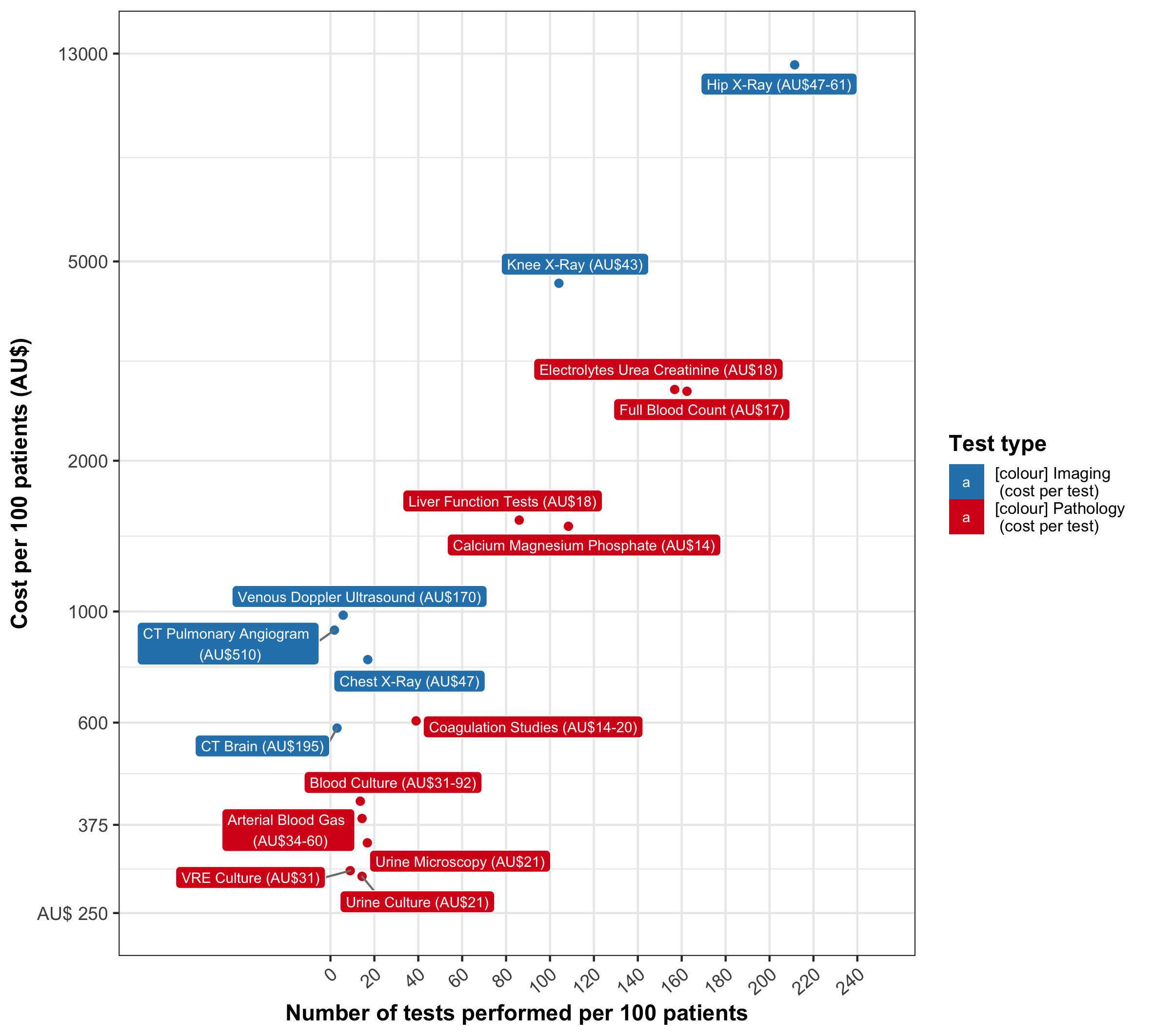

Supplement: Supplementary file 5 — Additional file 5. Cost and number of tests performed. Relationship between cost and number of tests performed (per 100 patients). [file 12913_2020_5892_MOESM5_ESM.png]
