# Supplementary material for: Cost of investigations during the acute hospital stay following total hip or knee arthroplasty, by complication status
Source: BMC Health Serv Res. 2020 Nov 12;20:1036. doi: 10.1186/s12913-020-05892-1 (PMC7659097; doi:10.1186/s12913-020-05892-1)
Supplement: Supplementary file 6 — Additional file 6. Mean number of tests by complication status. Mean number (SD) of imaging and pathology tests per 100 patients by complication status. [file 12913_2020_5892_MOESM6_ESM.docx]

Mean number (SD) of imaging and pathology tests per 100 patients by complication status.

|  | **No complications**  N=405 | **Minor only**  N=73 | **Major, at least one**  N=22 | **p-value** |
| --- | --- | --- | --- | --- |
| **Imaging** |  |  |  |  |
| Knee x-ray  (per 100 TKA patients) | 102 (12.7) | 115 (41.5) | 118 (40.5) | <0.001 |
| Hip x-ray (per 100 THA patients) | 203 (17.2) | 210 (30.1) | 245 (121) | 0.004 |
| Venous doppler ultrasound | 4.20 (20.1) | 12.3 (37.1) | 13.6 (35.1) | 0.009 |
| CT pulmonary angiogram | 0.49 (7.02) | 4.11 (20.0) | 18.2 (39.5) | <0.001 |
| Chest x-ray | 8.89 (31.0) | 37.0 (67.7) | 100 (138) | <0.001 |
| CT brain | 0.49 (7.02) | 15.1 (43.0) | 9.09 (29.4) | <0.001 |
| **Pathology** |  |  |  |  |
| Electrolytes urea creatinine | 131 (78.2) | 262 (286) | 282 (208) | <0.001 |
| Full blood count | 137 (86.3) | 266 (267) | 282 (199) | <0.001 |
| Liver function tests | 72.1 (59.6) | 142 (167) | 155 (141) | <0.001 |
| Calcium magnesium phosphate | 90.6 (67.2) | 170 (190) | 232 (208) | <0.001 |
| Coagulation studies | 28.6 (98.9) | 76.7 (203) | 105 (170) | <0.001 |
| Blood culture | 6.42 (30.8) | 41.1 (84.7) | 54.5 (91.2) | <0.001 |
| Arterial blood gas | 5.93 (28.4) | 23.3 (80.8) | 141 (259) | <0.001 |
| Urine microscopy | 8.15 (32.4) | 54.8 (81.7) | 50.0 (74.0) | <0.001 |
| VRE culture | 5.19 (29.8) | 20.5 (66.6) | 40.9 (73.4) | <0.001 |
| Urine culture | 6.91 (27.3) | 46.6 (72.8) | 45.5 (67.1) | <0.001 |

Abbreviations: TKA, total knee arthroplasty; THA, total hip arthroplasty; CT, computed tomography; VRE, vancomycin-resistant enterococci.
