# Supplementary material for: Cost of investigations during the acute hospital stay following total hip or knee arthroplasty, by complication status
Source: BMC Health Serv Res. 2020 Nov 12;20:1036. doi: 10.1186/s12913-020-05892-1 (PMC7659097; doi:10.1186/s12913-020-05892-1)
Supplement: Supplementary file 7 — Additional file 7. Mean cost of tests by complications status. Mean cost (SD) of imaging and pathology tests per patient by complication status in AU$. [file 12913_2020_5892_MOESM7_ESM.docx]

Mean cost (SD) of imaging and pathology tests per patient by complication status in AU$.

|  | **No complications**  N=405 | **Minor only**  N=73 | **Major, at least one**  N=22 | **p-value** |
| --- | --- | --- | --- | --- |
| **Imaging** |  |  |  |  |
| Knee x-ray  (per TKA patient) | 44.1 (5.5) | 50.1 (18.0) | 53.3 (22.6) | <0.001 |
| Hip x-ray (per THA patient) | 118 (24.8) | 124 (24.5) | 138 (70.6) | 0.137 |
| Venous doppler ultrasound | 7.11 (34.0) | 20.9 (62.8) | 23.1 (59.5) | 0.009 |
| CT pulmonary angiogram | 2.52 (35.8) | 21.0 (102) | 92.7 (201) | <0.001 |
| Chest x-ray | 4.19 (14.6) | 17.4 (31.9) | 47.2 (65.1) | <0.001 |
| CT brain | 0.96 (13.7) | 29.8 (84.5) | 18.6 (58.7) | <0.001 |
| **Pathology** |  |  |  |  |
| Electrolytes urea creatinine | 23.2 (13.8) | 46.3 (50.6) | 49.9 (36.9) | <0.001 |
| Full blood count | 23.3 (14.6) | 45.0 (45.3) | 47.8 (33.8) | <0.001 |
| Liver function tests | 12.8 (10.5) | 25.2 (29.6) | 27.4 (24.9) | <0.001 |
| Calcium magnesium phosphate | 12.4 (9.17) | 23.2 (25.9) | 31.6 (28.4) | <0.001 |
| Coagulation studies | 4.45 (15.3) | 11.8 (30.5) | 16.4 (27.0) | <0.001 |
| Blood culture | 1.97 (9.47) | 12.6 (26.0) | 16.8 (28.0) | <0.001 |
| Arterial blood gas | 1.81 (8.24) | 6.15 (17.5) | 34.0 (59.7) | <0.001 |
| Urine microscopy | 1.67 (6.65) | 11.3 (16.8) | 10.3 (15.2) | <0.001 |
| VRE culture | 1.75 (10.1) | 6.93 (22.5) | 13.8 (24.8) | <0.001 |
| Urine culture | 1.42 (5.61) | 9.57 (15.0) | 9.34 (13.8) | <0.001 |

Abbreviations: TKA, total knee arthroplasty; THA, total hip arthroplasty; CT, computed tomography; VRE, vancomycin-resistant enterococci.
